# Clinical features and predictors of mortality in admitted patients with community- and hospital-acquired legionellosis: A Danish historical cohort study

**DOI:** 10.1186/1471-2334-10-124

**Published:** 2010-05-21

**Authors:** Sanne Jespersen, Ole S Søgaard, Henrik C Schønheyder, Michael J Fine, Lars Østergaard

**Affiliations:** 1 Department of Infectious Diseases, Aarhus University Hospital, Skejby, Brendstrupgaardsvej 100, 8200 Aarhus N, Denmark; 2 Department of Clinical Microbiology, Aarhus University Hospital, Aalborg, Mølleparkvej 8A, 9000 Aalborg, Denmark; 3 Center for Health Equity Research and Promotion, VA Pittsburgh Healthcare System, 7180 Highland Drive (151C-H), Pittsburgh, PA 15206-1206, USA; 4 Division of General Medicine, Department of Medicine, University of Pittsburgh, UPMC Montefiore Hospital, Suite W933, PA 15213, USA

## Abstract

**Background:**

Legionella is a common cause of bacterial pneumonia. Community-acquired [CAL] and hospital-acquired legionellosis [HAL] may have different presentations and outcome. We aimed to compare clinical characteristics and examine predictors of mortality for CAL and HAL.

**Methods:**

We identified hospitalized cases of legionellosis in 4 Danish counties from January 1995 to December 2005 using the Danish national surveillance system and databases at departments of clinical microbiology. Clinical and laboratory data were retrieved from medical records; vital status was obtained from the Danish Civil Registration System. We calculated 30- and 90-day case fatality rates and identified independent predictors of mortality using logistic regression analyses.

**Results:**

We included 272 cases of CAL and 60 cases of HAL. Signs and symptoms of HAL were less pronounced than for CAL and time from in-hospital symptoms to legionellosis diagnosis was shorter for CAL than for HAL (5.5 days vs. 12 days p < 0.001). Thirty-day case fatality was 12.9% for CAL and 33.3% for HAL; similarly 90-day case fatalities in the two groups were 15.8% and 55.0%, respectively. In a logistic regression analysis (excluding symptoms and laboratory tests) age >65 years (OR = 2.6, 95% CI: 1.1-5.9) and Charlson comorbidty index ≥2 (OR = 2.7, 95% CI: 1.1-6.5) were associated with an increased risk of death in CAL. We identified no statistically significant predictors of 30-day mortality in HAL.

**Conclusions:**

Signs and symptoms were less pronounced in HAL compared to CAL. Conversely, 30-day case fatality was almost 3 times higher. Clinical awareness is important for the timely diagnosis and treatment especially of HAL. There is a need for further studies of prognostic factors in order to improve the therapeutic approach to legionellosis and potentially reduce mortality.

## Background

Legionellosis is caused by exposure to the small intracellular gram-negative bacterium Legionella from water sources in the indoor or outdoor environment [1]. Two to fifteen percent of all hospitalizations for community-acquired pneumonias in Europe and North America are caused by Legionella and recent studies suggest that rates of legionellosis may be increasing [2]. The optimal antibiotic treatment of legionellosis has never been investigated in a randomized clinical trial, but most clinicians use either macrolides or fluoroquinolones [3,4] alone or combined with rifampicin [5].

The most common presentation of legionellosis is pneumonia which is often severe but almost any manifestation of the disease has been seen ranging from a mild self-limiting flu-like illness called Pontiac fever to any extrapulmonary affection to multi organ failure and death. Although no single finding in legionellosis is pathognomonic several findings are characteristic [6] such as relative bradycardia, hyponatriemia, elevation in serum creatinine kinase, diarrhea, confusion and impaired liver- and kidney-function [6-8].

Prognostic factors in legionellosis are not well described but delay in appropriate antibiotic therapy has been associated with increased mortality [9]. Other potential prognostic factors include: Delayed hospitalization, long duration of symptoms prior to ICU admission, high SAPS II or APACHE II score, and hyponatriemia [9-11]. Most previous studies were small and did not analyze prognostic factors according to whether legionellosis was community- or hospital-acquired [9-11].

We designed a 10-year population-based historical cohort study in four Danish counties with the following aims: 1) To evaluate differences between community-acquired and hospital-acquired cases of legionellosis [CAL and HAL]; 2) To compare 30- and 90-day case fatality for patients with CAL and HAL; 3) To assess predictors of 30-day mortality for these 2 forms of legionellosis. A better understanding of clinical presentation and prognostic factors for legionellosis may optimize our therapeutic approach and management of legionellosis, and thus reduce mortality and morbidity.

## Methods

### Study setting and population

We included patients ≥15 years of age hospitalized with legionellosis in Aarhus, Viborg, Ringkøbing, and North Jutland counties (total population approximately 1.6 million)[12] between January 1995 and December 2005. All county residents have access to universal tax-supported health care provided by general practitioners and public hospitals.

Since 1968, all residents in Denmark have been registered in the Civil Registration System and given a unique identification number, which is used in all national registries to identify that person. The Civil Registration System provides daily updated information on death and emigration.

### Diagnosis of legionella infection

Diagnostic tests for legionellosis, a notifiable disease in Denmark, were performed at Statens Serum Institut (SSI), Copenhagen and the regional departments of clinical microbiology. Within the surveillance system cases are classified as either definitive, presumptive or unlikely cases. According to Danish guidelines a definitive case is a patient with at least one of the following: 1) Culture isolation of Legionella species; 2) Detection of Legionella antigen in urine with a level of antigen >10 aU/ml; 3) Fourfold rise or fall of immunofluorescence titres against L. pneumophila serogroup 1, 3 or 6 obtained with paired serum specimen, provided a titer of at least 1:128 was obtained; 4) Detection of L. pneumophila DNA by PCR in a sample from the lower airways or a Legionella antibody titer >1:256 against L. pneumophila serogroup 1, 3 or 6 combined with Legionella urinary antigen test >5 aU/ml and <10 aU/ml, or 5) Positive Legionella PCR and Legionella antibody titer >1:256 against L. pneumophila serogroup 1, 3 or 6. A presumptive case is defined as a patient with at least one of the following: 1) Legionella urinary antigen test >5 aU/ml and < 10 aU/ml; 2) Positive Legionella PCR; 3) An antibody titer >1:256 against L. pneumophila serogroup 1, 3 or 6; 4) A fourfold rise or fall of antibody titers with paired sera against L. pneumophila serogroup 2, 4 or 5 or against L. micdadei or L. bozemanii provided that a titer of at least 1:128 is reached; 5) A compatible history of legionellosis during an outbreak but without diagnostic tests being obtained, or 6) Any combination of diagnostic results that justifies a classification as a possible case. Only definitive and presumptive cases were included in the study as described previously [13]. To ensure that no cases of legionellosis were overlooked, the national surveillance system and local departments of clinical microbiology provided unique identification numbers for all patients with a positive diagnostic test. Only hospitalized cases were included since we did not have access to information from general practitioners for patients with legionellosis managed in the community setting. We defined legionellosis as hospital-acquired if a patient had been discharged within the preceding 10 days or if symptoms of legionellosis appeared >2 days after hospital admission. All other cases were defined as community-acquired. A case was defined as possibly travel-associated if the patient had been visiting a foreign country within 10 days of onset of symptoms.

### Baseline data collection

The following data were collected from patient medical records: Demographics, comorbidities, clinical signs and symptoms at baseline, date of onset of symptoms, blood biochemistry tests, chest X-rays, date of relevant diagnostic tests, results of diagnostic tests [13], time to diagnosis, admission to intensive care unit (ICU), complications (disseminated intravascular coagulation, rhabdomyolysis, myocardial infarction, cerebrovascular insult), antibiotic treatment, and length of hospital stay.

Comorbidity at the time of admission was assessed for each patient using the Charlson Comorbidity Index [CCI] which is defined using 19 major disease categories. The CCI is adapted and validated for use with hospital discharge ICD-9-CM codes and predicts short- and long-term mortality [14,15]. We used information from medical records to calculate a CURB65-score and pneumonia severity index (PSI) risk class as markers of illness severity [16,17] (missing values scored as 0). Baseline was defined as date of admission for community-acquired and date of onset of symptoms for hospital-acquired cases. Anti-legionella treatment was defined as any treatment with macrolides, fluoroquinolones, rifampicin and/or tetracyclines.

### Assesment of outcomes

The primary endpoint was death at 30 days from baseline. Vital status and date of death were obtained from the Danish Civil Registration System.

### Statistical analysis

We compared the demographics, clinical and laboratory features of CAL and HAL using χ^2 ^or Fisher's exact test as appropriate for categorical variables. Continuous variables were compared using two-sample t-test (normal distribution) or Mann-Whitney U-test (non-normal distribution).

To compare mortality between patients with CAL and HAL, we constructed Kaplan Meier plots of 30-day and 90-day mortality.

We examined predictors of 30-day mortality by logistic regression analyses for CAL and HAL separately. For CAL, variables identified as strong predictors of mortality (p-value < 0.1) were entered in a multivariate logistic regression model. For variables which were interdependent (i.e. CCI score and malignancy) only the strongest predictor was included. Variables deemed to be intermediary steps in the process leading to a fatal outcome (e.g. admission to ICU or intubation) were not included in the model. A second model was developed excluding laboratory tests since they as well could be seen as intermediary steps in the process leading to a fatal outcome. In this model we also excluded symptoms. Due to a low number of hospital-acquired cases multivariate analysis was only done for community-acquired cases.

Statistical analyses were performed using Stata 9.2 (Statacorp, Texas).

### Science ethics

This study was approved by the Danish Data Protection Agency (jr.-nr. 2006-41-6394) and the National Board of Health. According to Danish law no further approval was required at the time of collection of data.

## Results

We identified 370 cases of legionellosis, 22 cases who were never admitted to hospital and 16 cases with missing medical records were excluded. Of the 332 cases eligible for analysis 272 (81.9%) were community-acquired and 60 (18.1%) were hospital-acquired. For CAL 241 (88.6%) were reported to the Danish surveillance system whereas 57 cases of HAL (95%) were reported (p = 0.14). More cases of CAL (87.5%) were classified as definitive compared to HAL (80.0%) (p = 0.05).

### Comparison of baseline characteristics

The median age was 57 years (Interquartile range [IQR]: 50-70) among CAL and 67 years (IQR: 57-74) among HAL (p = 0.003) (table 1). Comorbidities were more common among HAL (91.7%) than among CAL (41.5%) (p < 0.001). Self-reported fever, headache, diarrhoea and temperature >39.5°C were more common among CAL than among HAL. CAL had higher median C-reactive protein level (285 mg/L), lower plasma sodium level (132 mmol/L), lower plasma potassium level (3.8 mmol/L) and higher hemoglobin (8.2 mmol/L) compared to HAL (161 mg/L (p < 0.001); 136 mmol/L (p < 0.001); 4.1 mmol/L (p < 0.001) and 7.3 mmol/L (p < 0.001) ) (Additional file 1). Chest X-ray was abnormal in 91.9% of community-acquired cases but only in 80.0% of hospital-acquired cases (p < 0.001). Duration of in-hospital symptoms to requesting a test for legionellosis (7.5 days versus 3 days, p < 0.001) and duration of in-hospital symptoms to diagnosis of legionellosis (12 days versus 5.5 days) (p < 0.001) were longer for HAL than for CAL.

**Table 1:** Baseline characteristics of patients with community-acquired (CAL) and hospital-acquired legionellosis (HAL).^*a*^

	CAL	HAL^*b*^	
**Characteristic**	**No. (%) (n = 272)^*c*^**	**No. (%) (n = 60)^*c*^**	**p-value^*d*^**

**Demographics**			
Male sex	181 (66.5)	33 (55.0)	0.09
Age, median years (IQR)	57 (50-70)	67 (57-74)	0.003*
Age group			0.002*
≤ 65 years	185 (68.0)	28 (46.7)	
> 65 years	87 (32.0)	32 (53.3)	
**Exposure**			
Associated with travelling to another country^*e*^	92 (33.8)	0 (0)	<0.001*
Exposure to air-conditioning	33 (12.1)	0 (0)	0.004*
Part of a possible outbreak of legionnaires' disease	2 (0.7)	1(1.7)	0.49
**Comorbid illnesses**			
Charlson Comorbidity Index			<0.001*
= 0	170 (62.5)	13 (21.7)	
= 1	61 (22.4)	12 (20.0)	
≥ 2	41 (15.1)	35 (58.3)	
Current smoker	147 (54.0)	17 (28.3)	0.002*
Current alcohol abuse	30 (11.0)	7 (11.7)	0.65
**Selected symptoms and signs**			
Temperature >39.5°C	143 (52.6)	7 (11.7)	<0.001*
Depressed consciousness	64 (23.5)	5 (8.3)	0.17
**X-ray findings**			
Abnormal chest X-ray	250 (91.9)	48 (80.0)	0.001*
Unilateral infiltrate	188 (69.1)	37 (61.7)	0.20
Bilateral infiltrates	54 (19.9)	9 (15.0)	0.36
**Severity of illness**			
Pneumonia severity index (PSI) risk class			0.74
I	19 (7.0)	3 (5.0)	
II	65 (23.9)	18 (30.0)	
III	76 (27.9)	15 (25.0)	
IV	95 (34.9)	19 (31.7)	
V	15 (5.5)	5 (8.3)	
CURB-65 score			0.62
O	119 (43.8)	21 (35.0)	
I	98 (36.0)	23 (38.3)	
II	39 (14.3)	12 (20.0)	
III	10 (3.7)	3 (5.0)	
IV	2 (0.7)	1 (1.7)	
**Diagnosis**			
Duration of in-hospital symptoms to relevant diagnostic test for legionellosis, median (IQR)	3 (1-5)	7.5 (4-15.5)	<0.001*
Duration of in-hospital symptoms to diagnosis of legionellosis, median (IQR)	5.5 (4-9)	12 (7-17)	<0.001*

a See additional file 1 for more baseline characteristics

b Defined as hospital-acquired if the patient had been admitted within the preceding 10 days or if the patient developed symptoms

of legionellosis more than 2 days after hospital admission.

c Totals may not add up to 100% due to missing values

d Differences between the groups were analyzed with χ^2 ^or Fisher's exact test as appropriate for categorical variables.

Continuous variables were compared using two-sample t-test (normal distribution) or Mann-Whitney U-test (non-normal distribution).

e Defined as possible travel-associated if the patient had travelled to a foreign country within 10 days of onset of symptoms.

IQR: Interquartile range.

* P-level < 0.05 is considered statistically significant.

For CAL and HAL, 48 and 6 patients (17.6% and 10.0%) received treatment with an anti-legionella antibiotic within 24 hours of baseline, respectively; 18 and 7 patients (6.6% and 11.7%, p = 0.24) received no anti-legionella antibiotic during admission (table 2). Among those receiving anti-legionella treatment, median delay from baseline was 2 days (IQR 1-4) days for CAL and 7 days (IQR 3-12) for HAL. For both CAL and HAL macrolide monotherapy was the most frequent anti-legionella treatment (32.7% and 30.0% of cases, respectively).

**Table 2:** Comparison of antibiotic therapy for CAL and HAL.

	CAL	HAL^*a*^	
**Variables**	**No. (%) of episodes**	**No. (%) of episodes**	**p-value^*b*^**

**Initial antibiotic therapy**			
Use of an anti-legionella antibiotic^*c*^	34 (12.5)	5 (8.3)	0.38
Use of >1 antibiotic (± anti-legionella antibiotics)	54 (19.9)	20 (33.3)	0.14
Penicillin monotherapy	144 (52.9)	16 (26.7)	<0.001*
**Anti-legionella antibiotic treatment**			
Anti-legionella antibiotic ≤ 24 hours^*d*^	48 (17.6)	6 (10.0)	x
Anti-legionella antibiotic > 24 hours^*d*^	201 (73.9)	42 (70.0)	x
No anti-legionella antibiotic during admission	18 (6.6)	7 (11.7)	0.24
**Delay of anti-legionella therapy **(median and IQR)			
Days from admission	2 (1-4)	X	x
Days from symptom onset	7 (5-11)	7 (3-12)	0.29
**Duration of anti-legionella therapy **(median and IQR)			
Died in hospital	7 (2-13)	6 (2-13)	0.79
Discharged alive	21 (14-23)	20 (13-27)	0.65
**Antibiotics used during admission**			
Macrolide monotherapy^*e*^	87 (32.0)	18 (30.0)	0.97
Fluoroquinolone monotherapy^*f*^	4 (1.5)	3 (5.0)	0.07
Macrolide + Rifampicin	39 (14.3)	5 (8.3)	0.26
Fluoroquinolone + Rifampicin	11 (4.0)	3 (5.0)	0.67
Macrolide + Fluoroquinolone	44 (16.2)	9 (15.0)	0.95
Macrolide + Fluoroquinolone + Rifampicin	67 (24.6)	15 (25.0)	0.77
No anti-legionella therapy	18 (6.6)	7 (11.7)	0.18
**Oral anti-legionella therapy only**			
Yes^*g*^	22 (8.1)	3 (5.0)	0.40
No	247 (90.8)	57 (95.0)	0.40

a A case was defined as hospital-acquired if the patient had been admitted within the preceding 10 days or if the patient developed

symptoms of legionellosis more than 2 days after hospital admission.

b Differences between the groups were analyzed with χ^2 ^or Fisher's exact test as appropriate for categorical variables.

Continuous variables were compared using two-sample t-test (normal distribution) or Mann-Whitney U-test (non-normal distribution).

c Anti-legionella antibiotics include macrolides, quinolones, rifampicin and tetracyclines.

d For community-acquired cases time from admission. For hospital-acquired cases time from beginning of symptoms.

No comparison between groups for these variables since baseline was defined in different ways.

e The macrolides were either erythromycin, clarithromycin, roxithromycin or azithromycin

with the most commonly used being intravenous erythromycin

f Primarily intravenous ciprofloxacin

g Most commonly used anti-legionella drug adminstered orally was roxithromycin

* P-level < 0.05 is considered statistically significant.

IQR: Interquartile range

### Comparison of mortality

**Figure 1:**
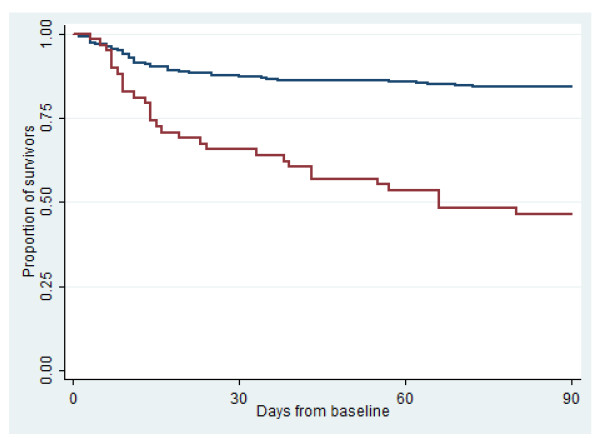
**Survival by community- or hospital-acquired infection. **Blue curve shows community-acquired cases, red curve hospital-acquired cases.

Thirty and 90-day case fatality were 12.9% and 15.8% for CAL, and 33.3% and 55.0% for HAL cases, respectively (figure 1 Survival by community- or hospital-acquired infection. Blue curve shows community-acquired cases, red curve hospital-acquired cases). One hundred-and-sixteen patients (42.6%) with CAL were admitted to intensive care unit [ICU] and 37 (61.7%) with HAL (p = 0.008) (table 3). Median length of stay was 12 and 10 days for CAL and HAL, respectively (p = 0.79).

**Table 3:** Comparison of outcomes for CAL and HAL.

	CAL	HAL^*a*^	
**Variables**	**No. (%)**	**No. (%)**	**p-value^*b*^**

**Admission to intensive care unit**	116 (42.6)	37 (61.7)	0.008*
Days in intensive care, median days (IQR)	12 (5-20)	10 (6-23)	0.79
Mechanical ventilation	93 (34.2)	32 (53.3)	0.06
Hemodialysis	43 (15.8)	18 (30.0)	0.01*
Inotropic support	86 (31.6)	29 (48.3)	0.02*
**Medical complications**			
Disseminated intravascular coagulation	9 (3.3)	3 (5.0)	0.53
Rhabdomyolysis	4 (1.5)	1 (1.7)	0.92
Myocardial infarction	5 (1.8)	4 (6.7)	0.04*
Cerebrovascular insult	5 (1.8)	0 (0)	0.29
**Outcome**			
30-day mortality^*c*^	35 (12.9)	20 (33.3)	x
90-day mortality^*c*^	43 (15.8)	33 (55.0)	x

a A case was defined as hospital-acquired if the patient had been admitted within the preceding 10 days or if the patient developed

symptoms of legionellosis more than 2 days after hospital admission.

b Differences between the groups were analyzed with χ^2 ^or Fisher's exact test as appropriate for categorical variables.

Continuous variables were compared using two-sample t-test (normal distribution) or Mann-Whitney U-test (non-normal distribution).

c For community-acquired cases time from admission. For hospital-acquired cases time from beginning of symptoms.

No comparison between groups for these variables since baseline was defined in different ways.

* P-level < 0.05 is considered statistically significant.

IQR: Interquartile range.

### Predictors of mortality for cal and hal

Among community-acquired cases no anti-legionella treatment was associated with a notable increased risk of death (Odds ratio [OR] = 11.5 (95% Confidence interval [CI]: 2.1-64.3) (table 4). Although not statistically significant, delay of anti-legionella treatment for more than 24 hours was associated with an increased risk of death (OR = 3.6, 95% CI: 0.8-15.6). Other variables associated with an increased risk of death in the unadjusted regression analysis can be seen in table 4. In a multivariate model including all variables with a p-value < 0.1 lymphocytosis (OR = 33.9, 95% CI: 2.1-553.5) and hyperbilirubinemia (OR = 7.3, 95% CI: 1.6-33.6) were independently associated with an increased risk of death; whereas hyponatriemia was associated with a decreased risk of death (OR = 0.2, 95% CI: 0.04-0.8) (p = 0.03). Median duration of time from in-hospital symptoms to diagnosis was shorter among patients with hyponatriemia compared with patients without, 5 days and 10 days, respectively (p < 0.001). Furthermore, 18% of patients with hyponatriemia received treatment with an anti-legionella antibiotic within 24 hours of admission compared to 10% of patients without hyponatriemia (p = 0.04). When replacing variables comprising PSI-score from the model (age, CCI score, confusion, hyponatriemia and blood urea nitrogen) with PSI score ≥3 this was independently associated with an increased risk of death (OR = 5.9, 95% CI: 1.2-28.9), whereas CURB65-score ≥ 3 was not an independent predictor of death OR = 1.9 (95% CI: 0.4-8.9) (p = 0.40). In a model that did not include symptoms and laboratory tests age >65 years (OR = 2.6, 95% CI: 1.1-5.9) and Charlson Comorbidity Index ≥2 (OR = 2.7, 95% CI: 1.1-6.5) were independent predictors of death (table 5).

**Table 4:** Univariate predictors of 30 day mortality in patients with CAL and HAL.^*a*^

	*CAL*		*HAL*^*b*^	
***Predictor variables by category***	***Odds ratio (95% CI)***	***P-value***	***Odds ratio (95% CI)***	***P-value***

*Variables known at the time of patient presentation*				
**Demographics**				
Age > 65 years	3.9 (1.9-8.1)	<0.001*	0.7 (0.2-2.0)	0.46
Case associated with travelling to another country	0.4 (0.1-0.9)	0.03*	x	x
**Comorbid illnesses**				
Any comorbidity^*c*^	2.4 (1.1-4.9)	0.02*	2.2 (0.2-21.5)	0.49
Immunosuppression	6.3 (2.7-14.8)	<0.001*	1.5 (0.5-4.6)	0.48
Malignancy	4.7 (1.6-13.9)	0.005*	2.1 (0.7-6.9)	0.20
Renal insufficiency	7.2 (1.4-37.1)	0.02	0.4 (0.04-3.4)	0.38
Charlson Comorbidity Index ≥ 2	3.7 (1.7-8.3)	0.001*	1.5 (0.5-4.6)	0.48
Current smoker	0.5 (0.2-1.1)	0.09	0.4 (0.1-1.6)	0.21
**Symptoms**				
Self-reported fever	0.4 (0.2-0.9)	0.03*	2.1 (0.7-6.6)	0.21
Dyspnoea	2.4 (1.1-5.1)	0.03*	0.6 (0.2-1.9)	0.42
Headache	0.1 (0.01-0.5)	0.01*	0.4 (0.05-4.3)	0.49
Confusion	1.9 (0.9-4.2)	0.01*	0.3 (0.03-2.5)	0.26
**Signs**				
Normal pulmonary stethoscopy	0.2 (0.1-1.02)	0.05	1.6 (0.3-8.4)	0.56
**Laboratory tests**				
Lymphocytosis, lymphocyte count >3.5 cells 10^9^/L	6.7 (0.9-49.6)	0.06	2.6 (0.1-45.4)	0.52
Bilirubin >23 mikromol/L	8.3 (2.9-24.1)	<0.001*	1.0 (0.3-4.1)	0.96
Creatinine >115 mikromol/L	2.5 (1.2-5.2)	0.01*	0.7 (0.2-2.6)	0.57
Blood urea nitrogen >7.5 mmol/L	4.2 (1.3-13.3)	0.02*	0.9 (1.1-5.6)	0.89
Hyponatremia, sodium <128 mmol/L	0.3 (0.1-1.0)	0.049*	0.6 (0.1-2.6)	0.49
Hypercapnia, PaCO2 >6 kPa	4.4 (0.97-19.9)	0.06	1.6 (0.3-10.1)	0.60
Atrial fibrillation at admission	2.6 (1.01-6.9)	0.048*	1.2 (0.3-5.1)	0.77
**X-ray findings**				
Unilateral infiltrate on chest x-ray	0.8 (0.4-1.6)	0.46	0.4 (0.1-1.2)	0.09
**Severity of illness**				
PSI risk class >2	9.1 (2.1-38.7)	<0.01*	2.2 (0.7-2.7)	0.20
CURB65 risk class >2	3.6 (1.0-12.8)	0.045*	0.6 (0.1-6.3)	0.68
				
*Variables known later during admission*				
**Treatment for legionellosis**				
Treatment with anti-legionella antibiotics				
<24 hours from baseline^*d*^	1 (ref.)		1 (ref.)	
>24 hours from baseline^*d*^	3.6 (0.8-15.6)	0.09	0.4 (0.1-2.0)	0.24
No treatment with anti-legionella antibiotics during admission	11.5 (2.1-64.3)	<0.01*	6.0 (0.4-85.2)	0.19

a For community-acquired cases 30 days from admission. For hospital-acquired cases 30 days from onset of symptoms

b A case was defined as hospital-acquired if the patient had been admitted within the preceding 10 days or if the patient developed

symptoms of legionellosis more than 2 days after hospital admission.

c Immunosuppression, malignancy, chronic obstructive pulmonary disease, ischaemic heart disease, congestive heart failure, diabetes mellitus

or renal insufficiency.

d For community-acquired cases time from admission. For hospital-acquired cases time from beginning of symptoms.

* P-level < 0.05 is considered statistically significant

**Table 5:** Independent predictors of 30 day mortality in patients with CAL.^*a*^

	*CAL*	
***Predictor variables by category***	***Odds ratio (95% conf. interval)***	***P-value***

**Demographics**		
Age > 65 years	1.3 (0.5-3.7)	0.60
Case associated with travelling to another country	0.4 (0.1-1.4)	0.16
**Comorbid illnesses**		
Charlson Comorbidity Index ≥ 2	2.8 (0.9-8.5)	0.08
Current smoker	0.7 (0.2-2.0)	0.46
**Symptoms**		
Self-reported fever	0.7 (0.2-2.3)	0.51
Dyspnoea	1.6 (0.6-4.3)	0.40
Headache	0.1 (0.01-1.03)	0.05
Confusion	3.0 (0.98-9.2)	0.06
**Signs**		
Normal pulmonary stethoscopy	0.3 (0.05-1.6)	0.16
**Laboratory tests**		
Lymphocytosis, lymphocyte count >3.5 cells 10^9^/L	33.9 (2.1-553.5)	0.01*
Bilirubin >23 mikromol/L	7.3 (1.6-33.4)	0.01*
Blood urea nitrogen >7.5 mmol/L	1.9 (0.4-8.5)	0.38
Hyponatriemia, sodium <128 mmol/L	0.2 (0.04-0.8)	0.03*
Hypercapnia, PaCO2 >6 kPa	2.4 (0.3-20.4)	0.42
Fibrillatrio atriorum at admission	2.4 (0.6-9.6)	0.22
**Treatment**		
Treatment with anti-legionella antibiotics < 24 hours from baseline^*b*^	0.3 (0.1-1.6)	0.16

**Model without symptoms and laboratory tests**		

	***CAL***	

***Predictor variables by category***	***Odds ratio (95% conf. interval)***	***P-value***

**Demographics**		
Age > 65 years	2.6 (1.1-5.9)	0.02*
Case associated with travelling to another country	0.5 (0.2-1.3)	0.15
**Comorbid illnesses**		
Charlson Comorbidity Index ≥ 2	2.7 (1.1-6.5)	0.03*
Current smoker	0.8 (0.3-1.9)	0.56
**Signs**		
Normal pulmonary stethoscopy	0.3 (0.1-1.2)	0.09
**Treatment**		
Treatment with anti-legionella antibiotics < 24 hours from baseline^*b*^	0.4 (0.1-1.7)	0.21

a For community-acquired cases 30 days from admission. For hospital-acquired cases 30 days from onset of symptoms

b For community-acquired cases time from admission. For hospital-acquired cases time from beginning of symptoms.

* P-level < 0.05 is considered statistically significant

Among hospital-acquired cases no variables were independently associated with a statistically significant increased risk of death though high odds ratios were found for the following variables: No treatment with anti-legionella antibiotics during admission, oxygen saturation <92%, ALAT >50 U/L, lymphocytosis, any comorbidity, PSI risk class >2, leucopenia, and anemia (table 4 and additional file 2).

## Discussion

In this historical population-based cohort study we found that HAL had a less distinctive clinical presentation and was associated with fewer abnormal biochemistry findings than CAL. Time from in-hospital symptoms to diagnosis was longer for HAL than for CAL, indicating a need for increased awareness of HAL. Legionellosis was associated with high mortality, particularly among hospital-acquired cases. Independent risk factors of death among community-acquired cases were age, comorbid conditions, lymphocytosis, and hyperbilirubinemia. Omission or delay of anti-legionella treatment was a risk factor for death among patients with CAL.

The strengths of our study include the population-based design, a laboratory-confirmed diagnosis in all cases, and a systematic review of patients' medical records. In Denmark there is equal access to a public health care system and reliable information on patients' date of death is available from the Central Person Registry.

Our study also had several limitations. Information about the time of initiation of antibiotic treatment was often imprecise, in most cases only the date of initiation was recorded and some patients who actually did not receive anti-legionella treatment within 24 hours of admission may have been misclassified. This potential bias would cause us to underestimate the effect of early initiation of anti-legionella treatment. Values needed for CURB65 and PSI scores were not available in all medical records, therefore we may have underestimated the prognostic accuracy of these two severity adjustment tools. Furthermore, the limited number of hospital-acquired cases reduced the power to detect variables significantly associated with increased mortality.

Only few studies have focused on differences between community-acquired and hospital-acquired cases of legionellosis [18,19]. Pedro-Botet et al. [19] compared 125 hospital-acquired cases and 33 community-acquired cases of legionellosis and found that younger age, smoking, cough, thoracic pain, and extrapulmonary manifestations were more prevalent in the community-acquired cases, while chronic lung disease, cancer, elevated blood creatinine values, and treatment with oxygen and corticosteroids were more prevalent among hospital-acquired cases in an unadjusted analysis. However, in the adjusted analysis only smoking and blood creatinine levels remained statistically significant. No differences were found in clinical outcome [19]. In our study, there were some notable differences between community-acquired and hospital-acquired cases. Hospital-acquired cases had less typical signs and symptoms of pneumonia compared to community-acquired cases. This might partly be explained by a more thorough medical history and examination when patients were admitted to the hospital compared to patients already being hospitalized - leading to information bias. Clinicians should be very observant of the diagnosis of legionellosis since 20% of hospital-acquired cases did not have abnormalities on chest x-rays at onset of symptoms. There was a marked delay of requesting testing for Legionella in HAL compared with CAL which again stresses the need for increased awareness and immediate access to diagnostics including PCR and urinary antigen testing. With this combination of tests a high diagnostic sensitivity is likely to be achieved [13].

Legionella-related mortality has decreased dramatically in the United States over the last decades. Benin et al. [20] examined 6757 confirmed cases of legionellosis from 1980-1998. During this period, case-fatality decreased from 46% to 14% for hospital-acquired cases, and from 26% to 10% for community-acquired cases. This improvement in outcome may be attributed to more prevalent use of diagnostic tests and increased use of broad-spectrum empirical antibiotic therapy for pneumonia [20]. The 30- and 90-days mortality-rate in our study was higher for both hospital-acquired and community-acquired cases than found by Benin et al. as well as in Pedro-Botet et al.'s study [19]. This could be explained to some extent by a more restrictive antibiotic policy in Denmark where the empirical antibiotic treatment for community-acquired pneumonia is penicillin with an exception made in some local guidelines for patients with a CURB65 score >2; addition of anti-legionella therapy is recommended for these patients. However, ascertainment bias must also be taken into account, i.e. that only the more severe cases of legionellosis tend to be diagnosed. A direct comparison with the studies of Benin et al. and Pedro-Botet et al. is not possible due to differences in case definitions. The studies by Benin et al. and Pedro-Botet et al. only included patients with Legionella pneumonia whereas we included all patients with legionellosis. Since patients with Pontiac fever have a lower risk of dying, mortality in our study would probably have been even higher if we had excluded these patients from our analyses.

Among community-acquired cases in our study, treatment with an anti-legionella drug within 24 hours of admission was associated with a decreased risk of death, however, this did not reach statistical significance. The lack of precision was primarily due to the limited sample size. Delay of anti-legionella therapy has found to be associated with an increased risk of death in other studies. A study of Heath et al. [9] of 39 serologically confirmed cases of legionellosis showed that the median delay in erythromycin therapy was 6 days for survivors and 11 days in those who died. Gacouin et al. [10] showed in a study of 43 cases with severe legionellosis that fluoroquinolone administration within 8 hours of ICU admission was associated with a reduced mortality (OR = 0.16; 95% CI: 0.03-0.96). In our study delayed treatment with anti-legionella antibiotics did not have a statistically significant impact on the risk of death among hospital-acquired cases. One contributing factor could be confounding by indication, i.e. that the most severely ill patients tended to receive treatment earlier.

A limited number of studies have focused on prognostic factors in legionellosis. Marston et al. [21] found that the likelihood of death from Legionella pneumonia was increased in patients who were elderly or male; this was also true for patients with hospital-acquired infection, renal disease, malignancy, immunosuppression, and for those with Legionella pneumophila serogroup 6. Except from the sex-difference in mortality these findings were in accordance with ours. Other studies of prognostic factors have only been done among patients with severe legionellosis requiring admission to ICU. Gacouin et al. [10] found that a SAPS II score >46, more than 5 days duration of symptoms prior to ICU admission, and intubation were associated with increased mortality in patients with legionellosis in the ICU but some of these factors are likely to be an integral part of the pathways which leads from the infection to the outcome and thus do not qualify as confounders in the statistical modeling. El-Ebiary et al. [11] also studied prognostic factors in patients with legionellosis requiring ICU admission and found that APACHE II score >15 at admission and serum sodium level <136 mmol/L were associated with a poor prognosis. Instead of APACHE II or SAPS II scores we used the PSI score and CURB65 scores and these severity assessment tools could predict patients with an increased risk of death among community-acquired cases in the univariate analysis even though some observations needed for calculation of a full score were not available. PSI risk class >2 remained an independent risk factor in the multivariate analysis. Surprisingly, we found hyponatriemia to be an independent predictor of a decreased risk of death. The association between hyponatriemia and legionellosis is well documented. Thus, increased awareness of legionellosis in patients with pneumonia and hyponatremia may have caused physicians to request diagnostic testing for legionellosis and to prescribe anti-legionella antibiotics as observed in our study which could explain the lower mortality in this group. We chose a statistical model without laboratory tests since abnormal values could be seen as intermediary steps in the process leading to a fatal outcome. Likewise, we also excluded symptoms from the model and found in consistence with the study done by Marston [21] that age and comorbidities were related to an increased risk of death.

## Conclusions

Signs and symptoms of hospital-acquired legionellosis were less pronounced than in community-acquired cases. However, the case fatality was almost 3 times higher. The prognosis for Danish patients with legionellosis was worse than reported in a recent US long-term study and we found a notable diagnostic delay in hospital-acquired cases. The study underlines that clinical awareness is important for the timely diagnosis and treatment especially of HAL. National guidelines for diagnosis and treatment of legionellosis could raise awareness of the disease and may improve quality of care. Studies of prognostic factors for legionellosis could further improve the therapeutic approach and potentially lead to a decrease in mortality.

## Competing interests

The authors declare that they have no competing interests.

## Authors' contributions

SJ collected all data, analyzed and interpreted data and drafted the manuscript.

OSS contributed to the analysis and interpretation of the data and helped draft the manuscript.

HCSN contributed to the analysis and interpretation of the data and revised the manuscript.

MJF contributed to the analysis and interpretation of the data and revised the manuscript.

LØ conceived of the study, and participated in its design and coordination.

All authors read and approved the final manuscript.

## Pre-publication history

The pre-publication history for this paper can be accessed here:

http://www.biomedcentral.com/1471-2334/10/124/prepub

## Supplementary Material

Additional file 1 **More baseline characteristics of the study population**. The additional file 1 contains a table showing baseline characteristics of the study population that are not shown in Table 1. Click here for file

Additional file 2 **Univariate predictors of 30 day mortality in patients with CAL and HAL (p > 0.1)**. The additional file 2 contains a table showing all the univariate predictors of 30 day mortality with a p-value > 0.1 in patients with community- and hospital-acquired legionellosis. Click here for file
